# Structural Damage, Visual Field Loss, and Quality of Life in Optic Disc Drusen: A Case–Control Study with Integrated Data-Driven Phenotypes

**DOI:** 10.3390/jcm15010061

**Published:** 2025-12-22

**Authors:** Alina Dumitriu, Bogdan Dumitriu, Luana Maria Gosman, Mihnea Munteanu

**Affiliations:** 1 Doctoral School, “Victor Babes” University of Medicine and Pharmacy Timisoara, 300041 Timisoara, Romania; alina.moatar@umft.ro; 2 Department of Ophthalmology, “Victor Babes” University of Medicine and Pharmacy Timisoara, 300041 Timisoara, Romania; luana.gosman@umft.ro (L.M.G.); mihnea.munteanu@umft.ro (M.M.)

**Keywords:** drusen, optic disk, visual fields, optic nerve, diseases, quality of life, case–control studies

## Abstract

**Background/Objectives:** Optic disc drusen (ODD) are an under-recognized cause of optic neuropathy, and the impact of structure–function damage on quality of life (QoL) is poorly defined. We compared systemic risk factors, ocular structure–function, and QoL in adults with ODD versus matched controls and identified determinants of impaired vision-related QoL. **Methods:** In a tertiary clinic, 60 adults with ultrasonography- or OCT-confirmed ODD were age- and sex-matched 1:1 to 60 controls without ODD. Retrospective clinical and imaging data (BCVA, RNFL thickness, standard automated perimetry) were combined with cross-sectional NEI VFQ-25 and EQ-5D-5L scores. **Results:** ODD patients more often had hypertension (51.7% vs. 31.7%, *p* = 0.026) and migraine (38.3% vs. 21.7%, *p* = 0.046). They showed worse BCVA (0.2 vs. 0.1 logMAR, *p* < 0.001), thinner RNFL (95.3 vs. 103.8 µm, *p* < 0.001), more depressed mean deviation (−4.7 vs. −1.3 dB, *p* < 0.001), and more frequent reproducible visual field defects (68.3% vs. 11.7%, *p* < 0.001). Vision-specific QoL was reduced (VFQ-25 composite 77.3 ± 11.4 vs. 89.7 ± 8.6, *p* < 0.001) and generic health status lower (EQ-5D utility 0.8 ± 0.1 vs. 0.9 ± 0.1, *p* < 0.001). In ODD, worse BCVA, more negative mean deviation and lower EQ-5D were independently associated with poorer VFQ-25 (model R^2^ = 0.57), while older age, thinner RNFL and migraine predicted visual field defects. **Conclusions:** ODD are associated with substantial visual field loss and clinically meaningful decrements in vision-related and generic QoL.

## 1. Introduction

Optic disc drusen (ODD) are acellular, calcified deposits located within the optic nerve head, typically anterior to the lamina cribrosa. They are thought to arise from impaired axonal metabolism within a congenitally narrow scleral canal in genetically predisposed individuals, leading to intra-axonal accumulation and subsequent extrusion and calcification of axoplasmic by-products [1]. Historically considered benign incidental findings, ODD are now increasingly recognized as a cause of structural optic neuropathy, with a high proportion of affected eyes demonstrating subclinical or symptomatic visual field (VF) loss [1]. Clinically, drusen may be superficial and obvious as refractile bodies, or deeply buried and detectable only with ancillary imaging such as B-scan ultrasonography or optical coherence tomography (OCT). Because buried ODD can mimic disc edema, it is a frequent diagnostic pitfall in patients investigated for suspected papilledema or raised intracranial pressure [1].

The reported prevalence of ODD in the general population ranges from approximately 0.3% to 2%, depending on age, ethnicity, and diagnostic method, with upper estimates around 2.4% when sensitive imaging techniques are used [1,2]. A recent systematic review and meta-analysis pooling data from multiple population-based and clinic-based cohorts confirmed that prevalence estimates cluster around 1–2% and forecast a substantial global increase in the number of individuals living with ODD as populations age [2]. In children, buried drusen predominate, with the Copenhagen Child Cohort 2000 Eye Study reporting ODD in approximately 0.4% of 11–12-year-olds and demonstrating that affected discs are on average smaller, with shorter axial length and a more crowded scleral canal, supporting a developmental-anatomical substrate for ODD formation [3]. Buried drusen may become more superficial and more heavily calcified with age, contributing to higher detection rates in adulthood and a dynamic structural evolution over the life course [1,2,3].

Modern multimodal imaging has substantially reshaped the understanding and clinical definition of ODD. The Optic Disc Drusen Studies (ODDS) Consortium has proposed OCT-based diagnostic criteria and positioned enhanced-depth imaging OCT (EDI-OCT) as the reference structural modality, with hyperreflective ovoid lesions within the prelaminar optic nerve head considered the hallmark of drusen [4]. These recommendations emphasize the importance of standardized scan protocols and careful distinction between ODD and other causes of optic disc elevation. Quantitative OCT studies have demonstrated that peripapillary retinal nerve fibre layer (RNFL) thinning is common in ODD and that thinner RNFL correlates with the presence, location, and extent of VF defects, underscoring the axonal nature of the injury [5]. Recent reviews highlight an expanded imaging phenotype that includes peripapillary hyperreflective ovoid mass-like structures (PHOMS), alterations in the ganglion cell–inner plexiform layer, and peripapillary microvascular changes on OCT angiography, which may represent early markers of damage and help differentiate pseudopapilledema from true optic disc edema [6]. Collectively, these advances have shifted ODD from a purely ophthalmoscopic curiosity to a well-defined structural optic neuropathy.

Functionally, ODD are strongly associated with VF defects. Classic cross-sectional series reported abnormal perimetry in the majority of eyes with visible or buried drusen, typically showing enlargement of the blind spot, arcuate and altitudinal scotomas, and generalized sensitivity loss, even when central visual acuity remains normal [7]. Longitudinal cohorts suggest that mean rates of standard automated perimetry (SAP) mean deviation decline are generally slow but not negligible, with a subset of “fast progressors” losing sensitivity at rates comparable to early glaucoma [8,9]. Structural–functional coupling has been demonstrated, with worse RNFL thinning associated with more negative VF mean deviation and more complex defect patterns [5]. Despite this, many patients remain asymptomatic until late stages because foveal function and Snellen acuity are relatively preserved, creating a potential mismatch between subjective visual complaints, central acuity, and the objective extent of peripheral field loss [1,5,7,8,9].

Beyond insidious field loss, ODD is linked to sight-threatening complications. Case series and registry-based studies have shown that ODD are overrepresented among young adults with non-arteritic anterior ischemic optic neuropathy (NAION), and that affected patients often carry conventional vascular risk factors such as hypertension, hyperlipidemia, or migraine [1,10]. The crowded, rigid optic nerve head environment created by drusen may predispose to ischemic events by impairing microvascular perfusion and amplifying mechanical susceptibility at the disc [1,6,10]. Peripapillary choroidal neovascularization and intraretinal or peripapillary hemorrhages, while less common, have also been described and may further compromise central or paracentral vision [1,6]. These observations reinforce the notion that, for a subset of patients, ODD constitutes not only an anatomical variant but a clinically meaningful optic neuropathy with a non-trivial risk of permanent visual disability.

Several systemic and ocular factors have been proposed as risk modifiers for ODD. Shorter axial length and hyperopic refractive error, which contribute to a small, crowded scleral canal, are more frequent among patients with drusen and in affected children [1,3]. Familial clustering and reports of autosomal dominant inheritance patterns point towards a genetic contribution, although specific causal loci remain incompletely defined [1]. In cohorts with ODD-associated NAION, traditional vascular risk factors such as arterial hypertension, obstructive sleep apnoea, and smoking are common, raising the possibility that systemic comorbidities influence not only the likelihood of ischemic complications but perhaps also the functional impact of drusen over time [10]. However, most prior work has focused on anatomical descriptors, imaging biomarkers, or VF outcomes, and has rarely integrated systemic risk profiling with patient-reported measures of visual functioning. As a result, the extent to which structural and perimetric abnormalities translate into everyday activity limitations and perceived health remains poorly characterized.

Quality of life (QoL) and patient-reported outcome measures (PROMs) are now considered key endpoints in chronic ophthalmic diseases, particularly in conditions dominated by field loss rather than loss of central acuity. The 25-item National Eye Institute Visual Function Questionnaire (NEI VFQ-25) is the most widely used vision-specific instrument; it captures vision-related functioning across domains such as general vision, near and distance tasks, peripheral vision, driving, social functioning, and mental health [11]. Population-based data from the Gutenberg Health Study provide age- and sex-stratified reference values and demonstrate that NEI VFQ-25 scores decline with worsening visual function across a range of ocular diseases, supporting its construct validity and usefulness for comparative research [12]. Generic health-status instruments such as the EuroQol EQ-5D, including its 5-level version (EQ-5D-5L), complement vision-specific tools by situating visual disability within an overall health-related QoL and utility framework, enabling cost–utility analyses and health-economic modelling [13]. At the same time, studies in low-vision populations suggest that EQ-5D is relatively insensitive to modest changes in visual function, highlighting the importance of using both disease-specific and generic instruments when assessing the impact of eye disease on patients’ lives [14]. In glaucoma, for example, faster rates of binocular VF loss are strongly associated with worse vision-related QoL as measured by NEI VFQ-25, underscoring the clinical relevance of perimetric progression beyond structural metrics alone [15].

Therefore, there is a clear need for integrated data linking structural damage, functional loss, systemic risk factors, and QoL, specifically in patients with ODD. To date, most ODD studies have emphasized diagnostic differentiation from papilledema, imaging biomarkers, or VF outcomes, while patient-centred outcomes have been largely overlooked [1,2,3,4,5,6,7,8,9,10]. The present retrospective–cross-sectional case–control study was designed to address this gap in a tertiary ophthalmology clinic. We aimed to (i) compare clinical–pathological characteristics and systemic risk factors between patients with ODD and matched controls without ODD; (ii) quantify the impact of ODD on vision-related and generic QoL using the NEI VFQ-25 and EQ-5D-5L; and (iii) explore within-ODD associations between structural damage, VF loss, and QoL, including multivariable predictors of impaired vision-related QoL.

## 2. Materials and Methods

### 2.1. Study Design, Setting, and Population

We conducted a single-centre observational study combining a retrospective chart review with a cross-sectional case–control survey of quality of life. The study took place at the Ophthalmology Clinic of a tertiary university hospital, which functions as a regional referral centre for optic nerve head anomalies and neuro-ophthalmic disorders. The retrospective component comprised consecutive patients with clinically suspected ODD evaluated between January 2010 and December 2024, in whom the diagnosis was confirmed by B-scan ultrasonography and/or characteristic EDI-OCT findings.

For the cross-sectional survey, we invited a subset of these patients who were alive, contactable, and able to attend an outpatient visit during the study inclusion window. Inclusion criteria for the ODD group were: age ≥ 18 years, ultrasonography- or OCT-confirmed ODD in at least one eye, availability of at least one reliable standard automated perimetry test, and sufficient language skills to complete questionnaires. Exclusion criteria included coexisting ocular conditions with major visual impact (advanced glaucoma, proliferative diabetic retinopathy, macular degeneration), neurologic diseases affecting vision, and significant cognitive impairment. Controls without ODD were recruited from the same clinic among patients presenting for refractive evaluation or cataract screening and were individually matched 1:1 to ODD cases by age (±3 years) and sex. Controls underwent the same imaging protocol to exclude ODD.

### 2.2. Retrospective Clinical and Imaging Data

Retrospective data were extracted from electronic medical records by trained investigators using a standardized form. Demographic variables included age and sex. Systemic risk factors captured were arterial hypertension, diabetes mellitus, hyperlipidemia, migraine, smoking status, and other vascular or autoimmune comorbidities when documented. Ocular variables included best-corrected visual acuity (BCVA) converted to logMAR, spherical equivalent refraction (D), axial length (mm) measured by optical biometry when available, and intraocular pressure (IOP, mmHg). For patients with ODD, we also recorded whether drusen were unilateral or bilateral and whether they were clinically visible or only detected on imaging.

In eyes with ODD, we additionally derived a semi-quantitative drusen burden score (0–3) based on combined ophthalmoscopic appearance, EDI-OCT, and B-scan ultrasonography. The optic disc was divided into four quadrants, and drusen were graded according to their number, confluence, and associated disc elevation as follows: 0 = minimal focal drusen confined to ≤1 quadrant without appreciable disc margin elevation; 1 = mild drusen burden with discrete lesions in 2 quadrants and/or mild disc elevation; 2 = moderate burden with drusen present in ≥3 quadrants and definite disc elevation or clustered drusen; and 3 = severe, confluent drusen occupying most of the disc with marked elevation and crowded disc margins. For descriptive analyses in the ODD group, we used this 0–3 scale as an ordinal measure of structural load. This score is exploratory and not a standardized or validated grading system, and its use is restricted to within-study correlation and clustering analyses.

Imaging data included peripapillary RNFL thickness obtained from spectral-domain OCT and qualitatively graded RNFL thinning (present/absent) based on colour-coded deviation maps. B-scan ultrasonography reports were reviewed for highly reflective calcific deposits consistent with ODD. Standard automated perimetry (24-2 strategy) provided global indices, of which we used mean deviation (MD, dB) as the primary functional measure. We defined a reproducible visual field defect as the presence of a consistent cluster of depressed test points on at least two reliable visual field tests according to standard reliability criteria. For the purposes of group-level comparisons, we analyzed data from the worse eye in each ODD patient, defined as the eye with more negative MD, because this eye is most likely to drive perceived functional limitation in daily life. In control participants, in whom both eyes typically had normal fields, a randomly selected eye was used. We acknowledge that this asymmetric eye selection may inflate between-group differences in structure–function measures and that our estimates should be interpreted as worst-eye contrasts rather than per-patient averages.

### 2.3. Prospective Quality-of-Life Assessment

The prospective component consisted of a structured visit during which participants completed validated questionnaires under supervision. Vision-related quality of life was assessed with the 25-item National Eye Institute Visual Function Questionnaire (VFQ-25), using the standard scoring algorithm to derive domain scores and a composite score ranging from 0 to 100, with higher scores indicating better self-reported visual function. Particular attention was paid to the general vision, near activities, and peripheral vision subscales, which we hypothesized would be most affected by ODD-related field loss.

Generic health-related quality of life was assessed using the EQ-5D-5L questionnaire, which evaluates five dimensions (mobility, self-care, usual activities, pain/discomfort, anxiety/depression) on five response levels. Health states were converted into a country-specific utility index (0–1 scale) and participants also rated their current overall health on the EQ visual analogue scale (EQ-VAS, 0–100). Questionnaires were self-administered in a quiet room, with research staff available to clarify wording without influencing responses. To minimize missing data, forms were checked for completeness before the participant left; any missing items were re-offered, and participants were free to decline answering sensitive questions.

### 2.4. Statistical Analysis

All analyses were performed using R software (version 4.3; R Foundation for Statistical Computing, Vienna, Austria). Continuous variables were summarized as mean ± standard deviation (SD) and categorical variables as counts and percentages. Group differences between ODD patients and controls were assessed using Welch’s *t*-tests for continuous variables (to account for potential inequality of variances) and χ^2^ tests for categorical variables; Fisher’s exact test was used when expected cell counts were <5. Before fitting multivariable models, we inspected pairwise correlation matrices and calculated variance inflation factors (VIFs) for candidate predictors to assess collinearity. No VIF exceeded commonly accepted thresholds for problematic multicollinearity, and all prespecified variables were retained in the final models. Two-sided *p*-values < 0.05 were considered statistically significant.

Within the ODD group, we compared patients with vs. without reproducible visual field defects using Welch’s *t*-tests for continuous variables. Pearson correlation coefficients (r) quantified associations between VFQ-25 composite score and clinical parameters (BCVA, MD, RNFL thickness, a semiquantitative drusen burden score, EQ-5D index, age). Correlation strength was interpreted using conventional thresholds, and *p*-values were derived from the t distribution with n−2 degrees of freedom. Finally, we constructed a multivariable linear regression model with VFQ-25 composite score as the dependent variable and age (per 10 years), BCVA (per 0.1 logMAR), MD (per 1 dB increase), EQ-5D index (per 0.1 unit), and migraine (yes/no) as predictors. Model fit was summarized by R^2^, and regression coefficients were reported with 95% confidence intervals (CI) and *p*-values. Assumptions of linearity, homoscedasticity, and normality of residuals were checked visually using residual plots and Q–Q plots.

K-means cluster analysis (k = 3) was then applied to the same standardized variables to identify latent clinical phenotypes; the number of clusters was chosen using the Calinski–Harabasz index. This unsupervised clustering represents an exploratory machine-learning approach to phenotype derivation rather than a predictive model. Principal component analysis (PCA) was performed on standardized BCVA, visual field MD, global RNFL thickness, VFQ-25 composite score, EQ-5D-5L utility, and drusen burden score. We examined solutions with two and three components, using the Kaiser criterion (eigenvalues > 1) and scree plot inspection to guide component retention. Because the third component explained only a small additional proportion of variance and did not reveal a distinct or clinically interpretable axis beyond PC1 and PC2, we present the two-component solution in the main text.

## 3. Results

Table 1 summarizes demographic and systemic characteristics of patients with optic disc drusen (ODD) compared with age- and sex-matched controls. Mean age and sex distribution were closely comparable between groups, indicating successful matching. However, ODD patients exhibited a higher burden of vascular and headache-related comorbidities: arterial hypertension was significantly more frequent in the ODD group (51.7% vs. 31.7%, *p* = 0.026), and migraine was almost twice as common (38.3% vs. 21.7%, *p* = 0.046). Hyperlipidemia also tended to be more prevalent in ODD, although this did not reach conventional statistical significance (46.7% vs. 31.7%, *p* = 0.092). The prevalence of diabetes mellitus and current smoking did not differ significantly between groups. Refractive and biometric parameters showed the expected anatomic profile of ODD: affected patients were more hyperopic (mean spherical equivalent + 0.9 D vs. −0.2 D, *p* = 0.001) and had shorter axial length (22.7 vs. 23.2 mm, *p* = 0.001), consistent with a smaller, more crowded scleral canal.

Table 2 details ocular structural and functional characteristics in ODD patients versus controls. Central visual acuity was mildly but significantly worse in ODD, with a mean BCVA of 0.2 ± 0.1 logMAR in the worse eye compared with 0.1 ± 0.1 logMAR in controls (*p* < 0.001), indicating that most patients retained relatively good central vision despite drusen. In contrast, peripapillary RNFL thickness was significantly reduced in the ODD group (95.3 ± 14.7 µm vs. 103.8 ± 9.6 µm, *p* < 0.001), and visual field mean deviation was substantially more depressed (−4.7 ± 3.8 dB vs. −1.3 ± 1.9 dB, *p* < 0.001), reflecting clinically relevant structural and functional damage. Intraocular pressure did not differ significantly between groups. Qualitative indices showed marked divergence: RNFL thinning was present in nearly two-thirds of ODD patients (63.3%) but in only 18.3% of controls (*p* < 0.001), and reproducible visual field defects were documented in 68.3% of ODD eyes compared with 11.7% of controls (*p* < 0.001).

Table 3 examines vision-related and generic quality of life outcomes in ODD patients and controls. Vision-specific QoL, as measured by the VFQ-25 composite score, was significantly lower in the ODD group (77.3 ± 11.4 vs. 89.7 ± 8.6, *p* < 0.001), indicating a meaningful impact of ODD on daily visual functioning. Domain-level analyses showed a consistent pattern: general vision, near activities, and peripheral vision scores were all significantly reduced in ODD (68.9 ± 14.2, 74.1 ± 13.8, and 69.4 ± 16.7, respectively) compared with controls (82.1 ± 11.7, 87.3 ± 9.9, and 85.2 ± 11.3; all *p* < 0.001), with the largest absolute differences observed for peripheral vision, consistent with the predominant field involvement. Generic health-related QoL was also impaired: the EQ-5D-5L utility index averaged 0.8 ± 0.1 in ODD versus 0.9 ± 0.1 in controls (*p* < 0.001), and EQ-VAS self-rated health scores were lower in ODD (71.4 ± 12.7 vs. 83.9 ± 10.3, *p* < 0.001).

Table 4 compares clinical, structural, functional, and QoL parameters within the ODD group according to the presence of reproducible visual field defects. Patients with VF defects were older on average (44.9 ± 10.7 vs. 38.6 ± 11.3 years, *p* = 0.041) and had worse central visual acuity (BCVA 0.2 ± 0.1 vs. 0.1 ± 0.1 logMAR, *p* = 0.001). Structurally, they showed more pronounced axonal loss, with thinner global RNFL thickness (90.8 ± 12.7 vs. 104.7 ± 12.1 µm, *p* < 0.001). Functionally, the difference in mean deviation was substantial (−6.7 ± 3.1 vs. −1.3 ± 1.7 dB, *p* < 0.001), delineating a more advanced field compromise in the defect group. Correspondingly, vision-related QoL was significantly lower in ODD patients with VF defects (VFQ-25 composite 72.4 ± 10.8 vs. 86.2 ± 8.7, *p* < 0.001), and generic health status was also reduced (EQ-5D-5L utility 0.7 ± 0.1 vs. 0.9 ± 0.1, *p* < 0.001).

Table 5 presents Pearson correlation coefficients between vision-related QoL (VFQ-25 composite score) and key clinical parameters in ODD patients. Worse central visual acuity (higher logMAR) was moderately associated with poorer QoL (r = −0.52, *p* < 0.001), while better visual field status (less negative MD) correlated positively with VFQ-25 (r = 0.47, *p* < 0.001), underscoring the functional relevance of both acuity and field integrity. Structural damage showed a weaker but still significant relationship: thinner RNFL was associated with lower QoL (r = 0.33, *p* = 0.008), and a higher drusen burden score correlated with worse VFQ-25 (r = −0.28, *p* = 0.026). The strongest single correlate of vision-related QoL was generic health status, with EQ-5D-5L utility showing a robust positive correlation (r = 0.61, *p* < 0.001), suggesting that systemic and psychosocial factors captured by EQ-5D add substantially to the variance in perceived visual functioning. Age showed a small, non-significant negative association (r = −0.18, *p* = 0.163), indicating that, within the studied age range, disease-related factors exert a greater influence on vision-related QoL than chronological age alone. By comparison, the correlation between RNFL thickness and VFQ-25 composite was modest (r = 0.33) and RNFL thickness was not included as a predictor in the final multivariable QoL model (Table 6), indicating that structural thinning, while relevant, is a weaker direct correlate of patient-reported disability than visual field loss or generic health status.

Table 6 reports the multivariable linear regression model identifying independent predictors of vision-related QoL (VFQ-25 composite score) in ODD patients. The model explained 57% of the variance in VFQ-25 (R^2^ = 0.57, overall *p* < 0.001), indicating good explanatory power. Older age was associated with lower QoL, with a 2.7-point decrease in VFQ-25 per 10-year increase (β = −2.7, 95% CI −5.1 to −0.3, *p* = 0.028). Worse central visual acuity independently predicted poorer QoL: each 0.1 logMAR worsening in BCVA was linked to a 3.9-point reduction in VFQ-25 (β = −3.9, 95% CI −6.5 to −1.3, *p* = 0.004). Visual field damage remained a significant determinant after adjustment, with each 1 dB improvement in MD associated with a 1.2-point increase in QoL (β = 1.2, 95% CI 0.6 to 1.8, *p* < 0.001). Generic health status showed a strong independent contribution, with a 0.1-unit gain in EQ-5D-5L utility corresponding to a 4.7-point higher VFQ-25 score (β = 4.7, 95% CI 2.3 to 7.1, *p* < 0.001). Migraine was associated with a trend towards lower QoL (β = −3.1, *p* = 0.058), suggesting a possible additional burden that did not quite reach statistical significance. There was no evidence of problematic multicollinearity among predictors in this model based on inspection of correlation matrices and VIFs.

Table 7 summarizes the multivariable logistic regression analysis for the presence of reproducible visual field defects in ODD patients. Increasing age was independently associated with a higher likelihood of VF defects, with an adjusted odds ratio (OR) of 1.6 per 10-year increase (95% CI 1.1–2.4, *p* = 0.018). Thinner RNFL strongly protected against normal fields, as each 10 µm increase in RNFL thickness was associated with a 40% reduction in the odds of having a VF defect (OR 0.6, 95% CI 0.3–0.8, *p* = 0.006), reinforcing the structural–functional link. Migraine was also an independent predictor (OR 2.1, 95% CI 1.1–4.4, *p* = 0.041), suggesting that headache-prone patients with ODD are more likely to exhibit objective field loss. Axial length, arterial hypertension, and spherical equivalent showed trends in the expected directions (shorter eyes, hypertensive status, and more hyperopic refraction associated with higher odds of VF defects), but did not reach statistical significance. The model’s pseudo-R^2^ of 0.34 and highly significant likelihood ratio test (*p* < 0.001) indicate a moderate ability to discriminate patients with and without reproducible field defects based on the included covariates.

Table 8 uses PCA to condense multiple correlated structural, functional, and patient-reported variables into interpretable latent dimensions. The first principal component (PC1) has an eigenvalue of 2.8 and explains 46.7% of total variance, with strong positive loadings from visual field mean deviation (0.8), RNFL thickness (0.7), VFQ-25 composite score (0.7), and EQ-5D utility (0.6), and strong negative loadings from recoded BCVA (−0.7) and drusen burden (−0.6). PC2 (eigenvalue 1.1, 18.3% variance) has a more nuanced pattern, with moderate positive loadings from drusen burden (0.6), RNFL (0.4), EQ-5D (0.4), and VFQ-25 (0.3), and modest negative loadings from mean deviation (−0.3) and BCVA (−0.1). A three-component solution was also inspected, but the third component accounted for only limited additional variance and lacked a clear, clinically meaningful pattern; therefore, it is not tabulated. This suggests a partial dissociation between anatomical drusen load and functional field performance: some patients with high drusen scores still maintain relatively preserved fields but differ in how these changes are experienced at the QoL level.

Table 9 introduces a more advanced, data-driven angle by using k-means clustering to identify latent clinical phenotypes among ODD patients. Three distinct clusters emerged. Cluster 1 (“Mild structural–high QoL”, *n* = 21) comprises patients with near-normal acuity (BCVA 0.1), relatively mild field loss (MD −2.3 dB), thickest RNFL (101.2 µm), and the lowest drusen burden (1.4). Their VFQ-25 (85.1) and EQ-5D (0.9) scores are highest, and fewer than half have a field defect (47.6%), with only 14.3% falling below the VFQ-25 < 70 threshold, representing a comparatively compensated phenotype.

Cluster 2 (“Damage–symptomatic”, *n* = 23) shows intermediate severity: BCVA averages 0.2, MD is more depressed (−5.2 dB), RNFL is thinner (94.3 µm), and drusen burden is moderate (2.1). Vision-related QoL is clearly reduced (VFQ-25 76.4), generic health slightly lower (EQ-5D 0.8), and roughly seven in ten patients have a visual field defect (69.6%). About one-third (30.4%) have VFQ-25 scores < 70, indicating clinically important functional impact.

Cluster 3 (“Advanced–QoL-impaired”, *n* = 16) contains the most vulnerable patients: they have the worst acuity (BCVA 0.3), most negative MD (−7.1 dB), thinnest RNFL (88.7 µm), and highest drusen burden (2.6). Correspondingly, VFQ-25 (67.9) and EQ-5D (0.7) are lowest; almost all have a field defect (93.8%), and over half (57.1%) fall below the VFQ-25 < 70 threshold.

**Figure 1:**
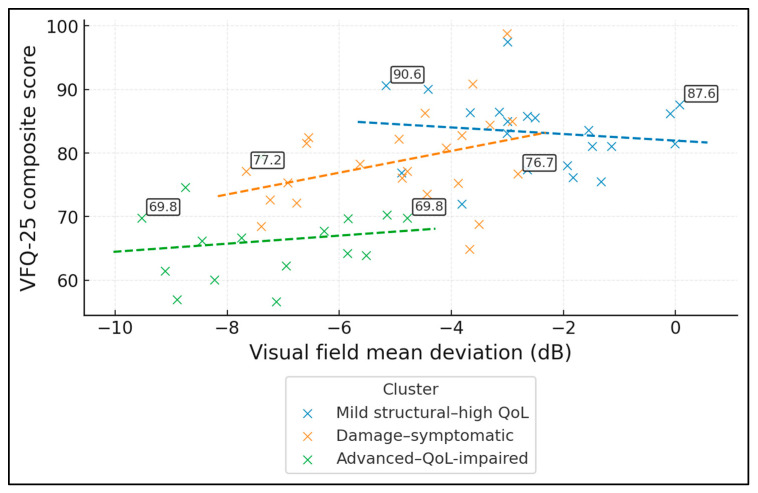
Relationship between visual field loss and vision-related QoL by phenotype (Scatterplot of VFQ-25 composite vs. visual field mean deviation, colour-coded by the three k-means phenotypes, with cluster-specific regression lines).

Figure 1 shows that vision-related QoL deteriorates as visual field damage increases, and that this relationship differs across the three data-driven ODD phenotypes. In the full ODD sample (*n* = 60), the Pearson correlation between mean deviation (MD, higher = better field) and VFQ-25 composite is r ≈ 0.60, indicating a moderately strong structure–function–QoL link. Cluster-level correlations are weaker but directionally similar: r ≈ −0.13 in the “mild structural–high QoL” group (*n* = 21), r ≈ 0.36 in the “damage–symptomatic” group (*n* = 23), and r ≈ 0.16 in the “advanced–QoL-impaired” group (*n* = 16). Regression lines highlight that for the same degree of MD loss, patients in the advanced cluster tend to report systematically lower VFQ-25 scores (often in the 60–70 range) compared with those in the mild cluster (typically 80–90).

**Figure 2:**
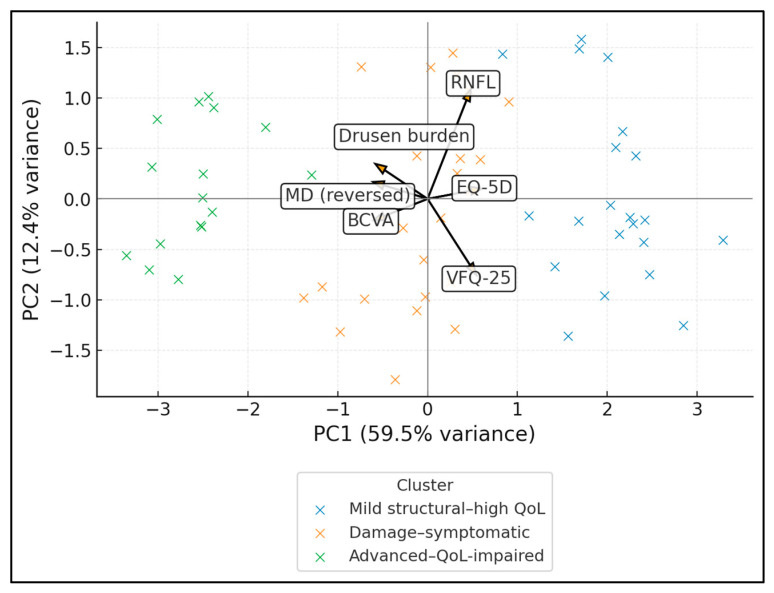
PCA biplot (structure–function–QoL integration).

The PCA biplot shows individual ODD patients in the PC1–PC2 plane, coloured by data-driven cluster, with arrows indicating loadings of each variable. PC1 explains 59.5% of the variance and represents a global “better structure/better function/better QoL” axis: MD (reversed), RNFL, VFQ-25 and EQ-5D load positively, while BCVA and drusen burden load negatively. PC2 (12.4% of variance) contrasts mainly RNFL (positive loading) with VFQ-25 (negative), suggesting a secondary dimension of structural–symptom discordance. Mild structural–high QoL patients cluster in the positive PC1 region, advanced–QoL-impaired cases in the negative PC1 region, and damage–symptomatic patients occupy an intermediate band (Figure 2).

**Figure 3:**
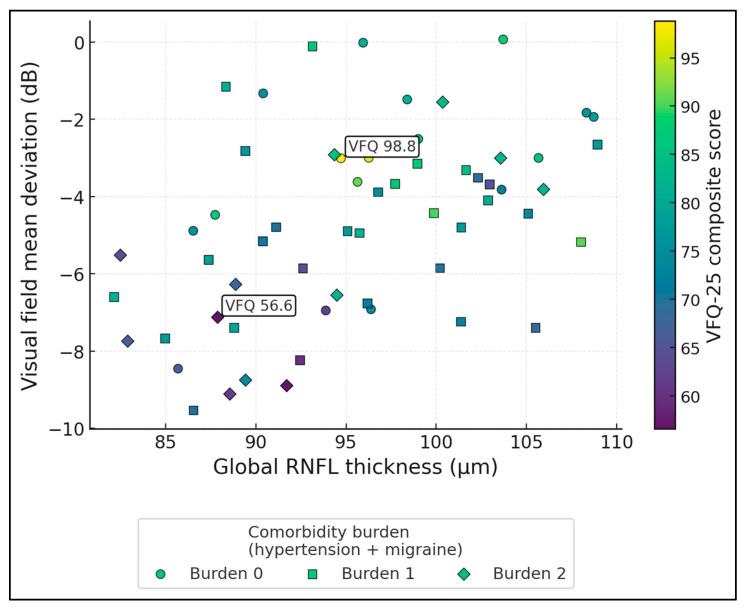
Structure–function–QoL triad with comorbidity burden.

Figure 3 maps global RNFL thickness (x-axis) against visual field mean deviation (y-axis), with point colour encoding VFQ-25 (range 56.6–98.8, mean 77.0) and marker shape indicating comorbidity burden (0, 1, or 2 = hypertension + migraine). RNFL and MD are moderately correlated (r ≈ 0.46), forming an upward trend where thinner RNFL corresponds to more negative MD. The two labelled points illustrate extremes: the “best” case (VFQ ≈ 98.8) lies in the high-RNFL, near-normal MD region, whereas the “worst” case (VFQ ≈ 56.6) sits in the low-RNFL, markedly depressed MD area. Higher-burden diamonds are over-represented in the lower-left quadrant, where both structure and function are poorest and colours shift toward lower VFQ values, visually linking systemic comorbidity with more severe structure–function damage and worse vision-related quality of life.

## 4. Discussion

### 4.1. Analysis of Findings

The present case–control data reinforce the view that ODD occur on a distinct systemic and anatomic background rather than representing an isolated incidental finding. The higher prevalence of arterial hypertension and migraine in the ODD group, together with more hyperopic refraction and shorter axial length, mirrors the “crowded disc” phenotype described in structural OCT series, in which small disc size and compact scleral canals are key substrates for drusen formation and related axonal crowding [16,17,18,19,20]. Quantitative EDI-OCT work by Malmqvist et al. demonstrated that larger drusen volume and more anterior location are associated with a smaller Bruch’s membrane opening and more pronounced disc crowding, consistent with the more hyperopic, short-axial-length profile we observed [19]. Similarly, Lee et al. found that eyes with type 2 ODD and more elevated lesions were older and showed worse mean deviation, supporting an interaction between age, disc anatomy, and functional vulnerability [20]. Our findings therefore extend previous anatomical descriptions by showing that this crowded optic disc milieu frequently coexists with systemic vascular and headache-related comorbidities, which may further reduce perfusion reserve at the optic nerve head.

Our multivariate framework also lends itself to future machine-learning (ML) applications. In the present study, we used k-means clustering as an unsupervised ML method to derive clinically interpretable ODD phenotypes based on standardized structural, functional and QoL variables. Principal component scores from the PCA could similarly be used as low-dimensional inputs to supervised ML algorithms (e.g., regularized logistic regression, random forests, gradient-boosted trees) aimed at predicting high-risk phenotypes such as the presence of reproducible VF defects or clinically important QoL impairment. However, the current sample size (60 ODD cases) is insufficient for robust training and external validation of such predictive models, and any ML-based risk scores would risk overfitting. We therefore regard our PCA and clustering results as exploratory proof-of-concept analyses that highlight the potential of integrating structure–function–QoL metrics into more advanced ML frameworks in larger, multicentre ODD cohorts.

Structurally and functionally, the magnitude of RNFL loss and VF impairment in our cohort is comparable to that reported in classic and contemporary ODD series. Roh et al. first quantified substantial RNFL thinning in ODD compared with normal controls using early OCT, with sectoral deficits corresponding to VF loss [16]. Katz and Pomeranz similarly showed that eyes with buried drusen frequently harbour arcuate or altitudinal VF defects co-localizing with RNFL defects on red-free photography, even when the disc appears only mildly anomalous [17]. In our study, global RNFL thickness was ~8 µm lower in ODD than in controls and reproducible VF defects were present in more than two-thirds of ODD eyes, figures that align with the 50–80% range of abnormal perimetry reported in earlier clinical cohorts [17,18,20]. Notably, the within-ODD comparison indicates that modest additional decrements in RNFL (≈14 µm) and MD (≈5 dB) separate patients with and without reproducible defects, echoing Lee et al.’s observation that RNFL thinner than ≈85 µm identifies eyes at highest risk for complex field loss [20]. Taken together, these data confirm that even nominally “mild” ODD are frequently accompanied by measurable axonal injury and functional compromise.

Beyond gross structure and fields, our results also converge with emerging evidence that ODD are associated with microvascular alterations at the optic nerve head. OCT-angiography studies have demonstrated focal capillary dropout and reduced peripapillary vessel density in eyes with ODD, particularly in regions overlying drusen, suggesting a local ischemic component to axonal injury [21,22]. Gaier et al. reported discrete zones of capillary rarefaction adjacent to drusen on OCT-A in patients with corresponding VF defects, while Cennamo et al. found lower peripapillary flow indices in ODD compared with controls, even after accounting for RNFL thickness [21,22]. Doppler ultrasound data also show disturbed central retinal artery hemodynamics in ODD, with reduced systolic and diastolic velocities and increased resistive indices relative to age-matched controls, reinforcing the concept of mechanical and vascular compromise around the disc [18]. Our finding that migraine independently predicts VF defects, together with trends for hypertension and hyperopia, is consistent with this microvascular vulnerability and with reports that ODD are over-represented among young patients with non-arteritic anterior ischemic optic neuropathy [10,18,19,20,21,22]. Clinically, this supports careful vascular risk assessment and counselling in ODD, particularly in older patients and those with headaches or systemic vascular disease.

To our knowledge, no prior ODD-focused study has systematically evaluated both vision-specific and generic QoL using NEI VFQ-25 and EQ-5D, so our data are best interpreted in the context of broader optic neuropathy literature. Psychometric studies of the NEI VFQ-25 have shown that it is highly responsive to differences in visual function and can detect clinically meaningful decrements even in moderate disease [23]. In glaucoma, van Gestel et al. and others demonstrated that progressive VF loss is independently associated with worse disease-specific and generic QoL, with decrements particularly marked once MD in the better eye falls beyond mild loss [24]. Jammal et al. reported that an MD of roughly −6 dB in the better eye already corresponds to a substantially higher probability of NEI VFQ-defined disability, even when visual acuity remains reasonable [25]. Our ODD group, with mean MD around −4.7 dB in the worse eye and a VFQ-25 composite in the high-70s, appears broadly comparable to early–moderate glaucoma in terms of QoL impact, despite largely preserved central acuity. The multivariable model showing independent effects of MD, BCVA and EQ-5D on VFQ-25 closely parallels glaucoma data in which both field loss and acuity are major determinants of vision-related QoL, and generic health measures capture additional variance beyond ocular metrics alone [23,24,25]. Importantly, the association between RNFL thickness and VFQ-25 was only of moderate magnitude, substantially weaker than that for MD or EQ-5D, reinforcing that structural metrics alone should not be used as surrogates for QoL and should instead be interpreted alongside functional testing and generic health measures.

Finally, the multivariate and cluster analyses in our cohort highlight substantial heterogeneity in how structural and functional damage translates into patient experience. Principal component analysis identified a dominant axis along which better MD, thicker RNFL, lower drusen burden, and higher VFQ-25 and EQ-5D scores co-varied, supporting a continuous spectrum from “compensated” to “advanced” ODD rather than discrete categories. The k-means phenotypes further refine this picture: a sizable subgroup with only mild structural damage and relatively preserved fields reported near-normal QoL, whereas an “advanced–QoL-impaired” cluster exhibited disproportionate functional and QoL burden for similar levels of anatomical change. Analogous dissociations between damage and disability have been documented in glaucoma, where some patients report pronounced QoL impairment at relatively modest VF loss and others remain functionally resilient until late stages [24,25]. Our data suggest that a similar pattern exists in ODD, with systemic health status and possibly migraine contributing to this variability. From a clinical standpoint, these findings argue for individualized risk stratification that integrates structural imaging, perimetry, and PROMs, rather than relying solely on disc appearance, and support targeted follow-up and counselling for patients clustering into high-risk, QoL-impaired phenotypes. Nevertheless, these findings may be influenced by patient-specific, environmental, healthcare-related, and study design factors; therefore, our results should be interpreted within the appropriate context [26,27,28,29,30,31,32,33].

From a practical standpoint, the present study can be reproduced in other neuro-ophthalmology clinics by following a simple set of steps. First, confirm the presence of ODD using EDI-OCT and/or B-scan ultrasonography, and exclude major coexisting ocular or neurological diseases with large independent effects on vision (e.g., advanced glaucoma, proliferative diabetic retinopathy, macular degeneration). Second, obtain standard structural and functional measurements in both eyes, including BCVA (logMAR), refraction, axial length, intraocular pressure, global peripapillary RNFL thickness from spectral-domain OCT, and 24-2 standard automated perimetry. Third, classify visual fields as normal or abnormal based on the presence of reproducible defects on at least two reliable tests, applying the same reproducibility criteria as defined in our Methods. Fourth, administer validated patient-reported outcome measures (NEI VFQ-25 and EQ-5D-5L) in the local language and score them using the standard algorithms. Finally, derive summary indices analogous to those used here (worse-eye MD and RNFL thickness, drusen burden score, VFQ-25 composite, EQ-5D-5L utility) to enable direct comparison with our regression, PCA and clustering models. Adhering to these rules allows clinicians to embed an ODD-specific structure–function–QoL evaluation pathway into routine care and to benchmark their local data against our case–control findings.

Nevertheless, this study shows that ODD should not be regarded as a benign incidental finding. Nearly seven in ten patients with ODD had reproducible visual field defects (68.3% vs. 11.7% in controls), with mean deviation depressed to −4.7 dB and accompanied by a ~12-point reduction in VFQ-25 composite score (77.3 vs. 89.7). Even though central acuity remained relatively good (mean BCVA 0.2 logMAR), patients reported significant difficulties with general, near and peripheral vision and lower generic health status (EQ-5D 0.8 vs. 0.9; EQ-VAS 71.4 vs. 83.9). These findings support systematic functional assessment (standard automated perimetry) and structural OCT (RNFL) in all patients with ODD, even when visual acuity is preserved. The strong link between QoL and both visual field status and EQ-5D underscores the value of integrating patient-reported outcome measures into routine follow-up. Identification of patients with thinner RNFL, more negative mean deviation, hypertension and migraine may help clinicians target closer surveillance, individualized counselling about activity limitations (e.g., driving, mobility in dim light), and early discussion of visual rehabilitation strategies in advanced phenotypes.

### 4.2. Study Limitations

This single-centre study was conducted in a tertiary referral clinic, which likely enriched the sample for more complex or symptomatic ODD and may limit generalisability to community or screening populations. As a consequence, the high prevalence of reproducible visual field defects (68.3% in ODD vs. 11.7% in controls) and the magnitude of QoL impairment we observed are best viewed as upper-bound estimates that probably exceed those in unselected individuals with incidentally detected ODD in primary or community care. The design combined retrospective chart review with a cross-sectional QoL survey, precluding causal inferences and limiting insight into longitudinal progression of structure, function and QoL. The sample size, although moderate (60 ODD, 60 controls), constrained the precision of multivariable estimates and of data-driven clustering, particularly in the smallest phenotype group. Analyses were based on the worse eye in the ODD group, which reflects patient-level impact but may obscure inter-eye asymmetry. In addition, the use of the worse eye in ODD and a randomly selected eye in controls introduces an asymmetry in eye selection that could exaggerate some between-group contrasts and contribute to Type I error. Our findings should therefore be interpreted as upper-bound estimates of structure–function differences and QoL decrements when comparing patients with clinically detected ODD in a tertiary setting with largely healthy controls. Self-reported instruments (VFQ-25, EQ-5D-5L) are subject to reporting bias and may be influenced by comorbid conditions not fully captured in the dataset. Finally, we did not include objective binocular performance measures (contrast sensitivity, real-world mobility tests) that could complement perimetry and PROMs in characterizing functional disability.

## 5. Conclusions

In this tertiary-clinic case–control study, adults with ODD exhibited a characteristic combination of a crowded optic nerve head, measurable structural damage, substantial visual field loss and clinically important reductions in both vision-specific and generic QoL. Hypertension and migraine were more prevalent among ODD patients, and within the ODD group, older age, thinner RNFL and migraine independently increased the odds of reproducible field defects, while worse acuity, more negative mean deviation and lower EQ-5D emerged as key determinants of impaired VFQ-25 scores (R^2^ = 0.57). Unsupervised clustering further highlighted heterogeneous phenotypes ranging from mildly affected, high-QoL patients to an advanced, QoL-impaired subgroup. Overall, these findings support reframing ODD as a structurally and functionally significant optic neuropathy with tangible patient-reported impact, and they argue for integrated management that combines structural and functional monitoring with systematic assessment of quality of life. Nevertheless, because our cohort was drawn from a tertiary referral clinic, the frequency of visual field defects and QoL impairment reported here likely overestimates the burden in the general population of individuals with ODD; however, the strong associations between structural damage, field loss, and patient-reported outcomes are expected to generalize across clinical settings.

## Figures and Tables

**Table 1:** Demographic and systemic characteristics of patients with optic disc drusen and controls.

Variable	ODD (*n* = 60)	Controls (*n* = 60)	*p*-Value
Age, years (mean ± SD)	42.7 ± 11.3	41.9 ± 10.7	0.692
Female sex, *n* (%)	35 (58.3%)	34 (56.7%)	0.853
Arterial hypertension, *n* (%)	31 (51.7%)	19 (31.7%)	0.026
Diabetes mellitus, *n* (%)	8 (13.3%)	5 (8.3%)	0.378
Migraine, *n* (%)	23 (38.3%)	13 (21.7%)	0.046
Hyperlipidemia, *n* (%)	28 (46.7%)	19 (31.7%)	0.092
Current smoking, *n* (%)	17 (28.3%)	13 (21.7%)	0.399
Spherical equivalent, D (mean ± SD)	0.9 ± 1.6	−0.2 ± 1.9	0.001
Axial length, mm (mean ± SD)	22.7 ± 0.8	23.2 ± 0.9	0.001

Data are presented as mean ± standard deviation (SD) or *n* (%), unless otherwise indicated. ODD, optic disc drusen; D, dioptres; SD, standard deviation.

**Table 2:** Ocular structural and functional characteristics.

Variable	ODD (*n* = 60)	Controls (*n* = 60)	*p*-Value
BCVA (worse eye), logMAR (mean ± SD)	0.2 ± 0.1	0.1 ± 0.1	<0.001
Global RNFL thickness, µm (mean ± SD)	95.3 ± 14.7	103.8 ± 9.6	<0.001
Visual field mean deviation, dB (mean ± SD)	−4.7 ± 3.8	−1.3 ± 1.9	<0.001
Intraocular pressure, mmHg (mean ± SD)	15.3 ± 2.7	14.7 ± 2.3	0.19
RNFL thinning present, *n* (%)	38 (63.3%)	11 (18.3%)	<0.001
Any reproducible VF defect, *n* (%)	41 (68.3%)	7 (11.7%)	<0.001

Data are presented as mean ± SD or *n* (%), unless otherwise indicated. ODD, optic disc drusen; BCVA, best-corrected visual acuity; RNFL, retinal nerve fibre layer; VF, visual field; SD, standard deviation.

**Table 3:** Vision-related and generic quality of life.

Outcome	ODD (*n* = 60)	Controls (*n* = 60)	*p*-Value
VFQ-25 composite score (0–100)	77.3 ± 11.4	89.7 ± 8.6	<0.001
VFQ-25 general vision	68.9 ± 14.2	82.1 ± 11.7	<0.001
VFQ-25 near activities	74.1 ± 13.8	87.3 ± 9.9	<0.001
VFQ-25 peripheral vision	69.4 ± 16.7	85.2 ± 11.3	<0.001
EQ-5D-5L utility index (0–1)	0.8 ± 0.1	0.9 ± 0.1	<0.001
EQ-VAS (0–100)	71.4 ± 12.7	83.9 ± 10.3	<0.001

Data are presented as mean ± SD. ODD, optic disc drusen; VFQ-25, 25-item National Eye Institute Visual Function Questionnaire; EQ-5D-5L, EuroQol 5-Dimension 5-Level instrument; EQ-VAS, EuroQol visual analogue scale.

**Table 4:** Within-ODD comparison: patients with vs. without reproducible visual field defects.

Variable	VF Defect (*n* = 41)	No VF Defect (*n* = 19)	*p*-Value
Age, years (mean ± SD)	44.9 ± 10.7	38.6 ± 11.3	0.041
BCVA (worse eye), logMAR	0.2 ± 0.1	0.1 ± 0.1	0.001
Global RNFL thickness, µm	90.8 ± 12.7	104.7 ± 12.1	<0.001
Visual field mean deviation, dB	−6.7 ± 3.1	−1.3 ± 1.7	<0.001
VFQ-25 composite score	72.4 ± 10.8	86.2 ± 8.7	<0.001
EQ-5D-5L utility index	0.7 ± 0.1	0.9 ± 0.1	<0.001

Data are presented as mean ± SD. ODD, optic disc drusen; VF, visual field; BCVA, best-corrected visual acuity; RNFL, retinal nerve fibre layer; VFQ-25, 25-item National Eye Institute Visual Function Questionnaire; EQ-5D-5L, EuroQol 5-Dimension 5-Level instrument; SD, standard deviation.

**Table 5:** Correlations between vision-related quality of life and clinical parameters in ODD patients.

Predictor (vs. VFQ-25 Composite)	Pearson r	*p*-Value
BCVA (worse eye), logMAR	−0.52	<0.001
Visual field mean deviation, dB	0.47	<0.001
Global RNFL thickness, µm	0.33	0.008
Drusen burden score (0–3)	−0.28	0.026
EQ-5D-5L utility index	0.61	<0.001
Age, years	−0.18	0.163

VFQ-25, 25-item National Eye Institute Visual Function Questionnaire; BCVA, best-corrected visual acuity; RNFL, retinal nerve fibre layer; EQ-5D-5L, EuroQol 5-Dimension 5-Level instrument; Drusen burden score (0–3): 0 = minimal focal drusen confined to ≤1 quadrant without disc elevation; 1 = mild drusen in 2 quadrants and/or mild elevation; 2 = moderate drusen in ≥3 quadrants with definite elevation; 3 = severe, confluent drusen occupying most of the disc with marked elevation.

**Table 6:** Multivariable predictors of vision-related quality of life (VFQ-25 composite) in ODD patients.

Predictor	β (Unstandardized)	95% CI	*p*-Value
Age (per 10-year increase)	−2.7	−5.1 to −0.3	0.028
BCVA (per 0.1 logMAR increase)	−3.9	−6.5 to −1.3	0.004
Visual field mean deviation (per 1 dB increase)	1.2	0.6 to 1.8	<0.001
EQ-5D-5L utility index (per 0.1 unit increase)	4.7	2.3 to 7.1	<0.001
Migraine (yes vs. no)	−3.1	−6.3 to 0.1	0.058

Outcome: VFQ-25 composite score. Linear regression model R^2^ = 0.57, overall *p* < 0.001; VFQ-25, 25-item National Eye Institute Visual Function Questionnaire; BCVA, best-corrected visual acuity; EuroQol 5-Dimension 5-Level instrument; CI, confidence interval; R^2^, coefficient of determination.

**Table 7:** Multivariable logistic regression for presence of reproducible visual field defect in ODD patients.

Predictor	Adjusted OR	95% CI	*p*-Value
Age (per 10-year increase)	1.6	1.1–2.4	0.018
Global RNFL thickness (per 10 µm increase)	0.6	0.3–0.8	0.006
Axial length (per 1 mm increase)	0.7	0.4–1.1	0.188
Arterial hypertension (yes vs. no)	1.8	0.9–3.7	0.103
Migraine (yes vs. no)	2.1	1.1–4.4	0.041
Spherical equivalent (per + 1 D)	1.3	0.9–1.9	0.126

Model pseudo-R^2^ = 0.34; likelihood ratio test *p* < 0.001. RNFL, retinal nerve fibre layer; OR, odds ratio; CI, confidence interval.

**Table 8:** Principal component analysis (PCA) of structural, functional, and QoL metrics in ODD patients.

Component	Eigen Value	Variance Explained (%)
PC1	2.8	46.7
PC2	1.1	18.3
Variable	PC1	PC2
BCVA (worse eye, logMAR *)	−0.7	−0.1
Visual field mean deviation (dB) †	0.8	−0.3
Global RNFL thickness (µm)	0.7	0.4
VFQ-25 composite score	0.7	0.3
EQ-5D-5L utility index	0.6	0.4
Drusen burden score (0–3)	−0.6	0.6

BCVA, best-corrected visual acuity; RNFL, retinal nerve fibre layer; VFQ-25, 25-item National Eye Institute Visual Function Questionnaire. * BCVA was recoded so higher values reflect worse acuity before PCA. † Mean deviation was sign-reversed so higher values indicate better field performance; Drusen burden score (0–3): 0 = minimal focal drusen confined to ≤1 quadrant without disc elevation; 1 = mild drusen in 2 quadrants and/or mild elevation; 2 = moderate drusen in ≥3 quadrants with definite elevation; 3 = severe, confluent drusen occupying most of the disc with marked elevation.

**Table 9:** Data-driven ODD phenotypes derived from k-means clustering on structural, functional, and QoL variables.

Cluster (Label)	*n*	BCVA (logMAR)	Visual Field MD (dB)	Global RNFL (µm)	Drusen Burden (0–3)	VFQ-25 Composite	EQ-5D Utility	Patients with VF Defect, %	Patients with VFQ-25 < 70, %
1—Mild structural–high QoL	21	0.1	−2.3	101.2	1.4	85.1	0.9	47.6	14.3
2—Damage–symptomatic	23	0.2	−5.2	94.3	2.1	76.4	0.8	69.6	30.4
3—Advanced–QoL-impaired	16	0.3	−7.1	88.7	2.6	67.9	0.7	93.8	57.1

MD, mean deviation; RNFL, retinal nerve fibre layer; VFQ-25, 25-item National Eye Institute Visual Function Questionnaire. Clustering performed on standardized BCVA, MD, RNFL, VFQ-25, EQ-5D, and drusen burden; k = 3 chosen by Calinski–Harabasz criterion; Drusen burden score (0–3): 0 = minimal focal drusen confined to ≤1 quadrant without disc elevation; 1 = mild drusen in 2 quadrants and/or mild elevation; 2 = moderate drusen in ≥3 quadrants with definite elevation; 3 = severe, confluent drusen occupying most of the disc with marked elevation.

## Data Availability

The data presented in this study are available on request from the corresponding author.
